# Challenges and Perspectives in the Study of Self-Incompatibility in Orchids

**DOI:** 10.3390/ijms222312901

**Published:** 2021-11-29

**Authors:** Xiaojing Zhang, Yin Jia, Yang Liu, Duanfen Chen, Yibo Luo, Shance Niu

**Affiliations:** 1 College of Horticulture, Hebei Agricultural University, Baoding 071001, China; zhangxiaojing702@163.com (X.Z.); 15930220597@139.com (Y.J.); lyang15502416083@163.com (Y.L.); chenduanfen@163.com (D.C.); 2 State Key Laboratory of Systematic and Evolutionary Botany, Institute of Botany, Chinese Academy of Sciences, Beijing 100093, China; 3 State Key Laboratory of North China Crop Improvement and Regulation, Hebei Agricultural University, Baoding 071001, China

**Keywords:** orchids, *Epidendroideae*, self-incompatibility, morphology, physiology, molecule

## Abstract

Self-incompatibility affects not only the formation of seeds, but also the evolution of species diversity. A robust understanding of the molecular mechanisms of self-incompatibility is essential for breeding efforts, as well as conservation biology research. In recent years, phenotypic and multiple omics studies have revealed that self-incompatibility in *Orchidaceae* is mainly concentrated in the subfamily *Epidendroideae*, and the self-incompatibility phenotypes are diverse, even in the same genus, and hormones (auxin and ethylene), and new male and female determinants might be involved in SI response. This work provides a good foundation for future studies of the evolution and molecular mechanisms of self-incompatibility. We review recent research progress on self-incompatibility in orchids at the morphological, physiological, and molecular levels, provide a general overview of self-incompatibility in orchids, and propose future research directions.

## 1. Introduction

Self-incompatibility (SI) refers to the inability of hermaphroditic angiosperms to self-pollinate, which promotes outcrossing or hybridization [1]. Self-pollination can reduce the genetic diversity of populations and increase the extinction rate of species, which is why self-pollination is often considered an ‘evolutionary dead-end’; consequently, some species have evolved mechanisms to avoid self-pollination and promote outcrossing (i.e., SI), [2,3]. SI plays a key role in maintaining high levels of genetic variation and improving the adaptability of species. The evolution of SI has been shaped by frequency-dependent selection and balancing selection [4,5].

Generally, SI is divided into two categories based on genetic characteristics: gametophytic self-incompatibility (GSI) and sporophytic self-incompatibility (SSI). SI is controlled by a single or multiple *S*-loci with a variety of *S*-haplotypes. The specific interactions between both male and female specificity determinants (*S*-determinants), which are tightly linked at the SI loci, induce SI responses. The key determinants and molecular mechanisms of SI in dicotyledons are well studied. Most studies have focused on Brassicaceae (SRK-based SSI systems), Solanaceae, Rosaceae, Plantaginaceae (*S*-RNase-based GSI systems), and *Papaveraceae* (S-glycoprotein-based GSI systems) [3,6,7,8,9,10]. Review has provided useful information on SI in dicotyledons [3]. By contrast, the molecular mechanisms of SI in monocotyledons remain largely unknown [3,11].

SI has been reported in more than 100 families and 39% of angiosperm species [12], there are approximately 11 families and 75 genera in monocotyledons [13]. SI has been documented over 750 species of *Orchidaceae*, the largest monocotyledons family [14,15], indicating that more species should be clarified. Recent studies have identified several structural mechanisms, phytohormones, and candidate genes involved in SI responses in orchids. In this review, we focus on recent research progress on orchid SI at the morphological, physiological, and molecular levels. We also discuss the diverse molecular mechanisms of SI and future research directions.

## 2. Morphological Types of SI in *Orchidaceae*

**Figure 1:**
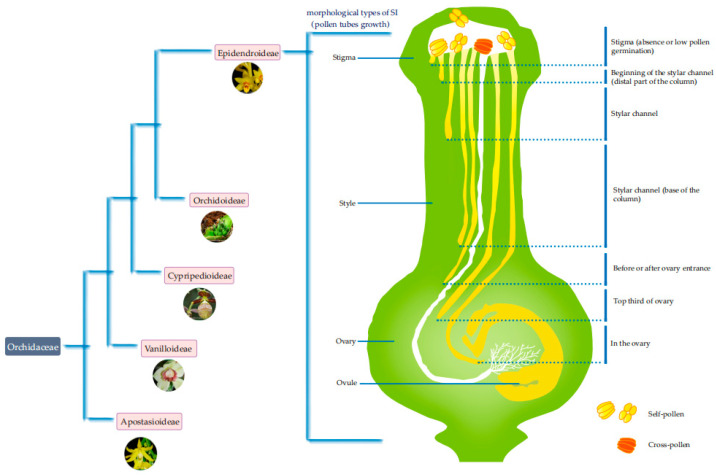
Diverse SI pollen tube morphologies exist in orchids in the subfamily *Epidendroideae*.

Many traits are used to measure the SI of orchids. Pollen tube growth, fruit set, and seed abortion rate are often used as the main indicators of SI, especially the growth of the pollen tube, as it is one of the most direct and important indicators of SI [16,17]. Although SI has been reported in various orchid groups, it is most common in a few groups, such as Dendrobiinae (i.e., *Dendrobium*) [18], *Pleurothallidinae* [19], *Oncidiinae* [14,20], *Malaxidinae* [21,22,23], *Laeliinae* [14,24], *Aeridinae* [25], *Angraecinae* [25], and *Neottieae* (i.e., *Epipactis*) [14], which all belong to the subfamily *Epidendroideae* (Figure 1). Here, we mainly review the pollen tube growth in orchid SI species.

### 2.1. Subtribe Dendrobiinae

Studies of the SI of the subtribe have focused on *Dendrobium*. *Dendrobium* is the second largest genus in *Orchidaceae* (second only to *Bulophyllum*) [26], with approximately 1450 species. It is a perennial epiphytic herb that is mainly distributed in tropical, subtropical, and temperate regions of Oceania and Asia [27].

By conducting more than 1700 pollination experiments on 61 species of *Dendrobium* [18], Johansen found that 44/61 (72%) of the species had wilted and yellowed ovaries and showed self-sterility after self-pollination. Among the remaining 17 fruit-producing species, the fruit ripening time and seed quality varied considerably. Analysis of the development of the self-pollenated tubes of *D. fameri* (SI) suggested that it was consistent with the GSI phenotype given that the pollen tubes enter the style (Table 1). This study indicated that some species of *Dendrobium* showed the GSI phenotype. Niu et al. [28] studied 26 representative species of *Dendrobium* and analyzed the pollen tube growth of 13 species (Table 1). Four kinds of pollen tube growth phenotypes were observed in self-incompatible species: (1) pollinia did not germinate; (2) the pollen tube stopped growing at the top of the style; (3) the pollen tube stopped growing at a specific position in the stylar channel, mostly at the upper third of the stylar channel; and (4) the pollen tube stopped growing in the upper third of the ovary. For example, the pollen tube of *D. densiflorum* stopped growing just before or after the style entrance one day after self-pollination (Table 1). The self-pollinated pollen tube of *D. chrysanthum* stopped growing at the upper third of the style three days after self-pollination (Table 1). The pollen tube of *D. lindleyi* stopped growing at the point before or just after the ovary entrance (Table 1) [28]. The growth of the pollen tube in more than half of the self-incompatible species stops in the style, and these species are distributed in different branches of *Dendrobium* phylogenetic tree [28,29], which is the main SI phenotype and consistent with the GSI phenotype. The diverse pollen germination and pollen tube growth phenotypes suggest that there might be more than one molecular mechanism of SI in *Dendrobium* species.

All these results show significant differences to those of molecular mechanisms known in other angiosperm families. There is a surprisingly high SI phenotype diversity in orchids, even in one genus, while there is only one SI phenotype described in other angiosperm families with their molecular mechanisms known, respectively. Furthermore, the emergence times of the SI phenotypes after self-pollination varies from species to species, mostly at three to five days, but even at two to three weeks, which much longer than that (mostly from tens of minutes to hours [35,36]) in other angiosperm families with their molecular mechanisms known. Therefore, investigation of more other orchids SI species is needed, which may reveal more SI phenotypes.

### 2.2. Subtribe Pleurothallidinae

SI analysis of *Restrepia*, belonging to clade B [19] of the *Pleurothallidinae*, revealed that 45% of the species are self-incompatible, and seed abortion is lower for cross-pollinated plants (both intraspecific and interspecific cross-pollination) than for self-pollinated plants. Pollen tubes fill the entire ovary after three weeks of cross-pollination, but the number of self-pollinated pollen tubes decreases significantly and stops growing in the upper third of the ovary, as is the case in the *R.* *brachypus* (Table 1) [16]. The genus *Acianthera* belongs to clade C [19] of the *Pleurothallidinae*. After self-pollination, the pollen tube growth of *A. fabiobarrosii* stops in the style (Table 1). Aside from the style, the ovary was found to be the second reaction site of SI, and it might be an extension of the SI reaction in the style [33].

In addition to pollen tubes, some *Pleurothallidinae* individuals can produce fruits with no seeds after self-pollination [19,34]. Seed abortion of *A. johannensis* after self-pollination may be caused by SI or by inbreeding depression [37]. What’s more, in *Anathallis sclerophylla*, *A. heterophylla*, and *A. rubens*, nearly all pollen grains do not germinate after self-pollination (Table 1), which is a typical SSI phenotype [32], but reciprocal crosses are needed for further verification.

The characteristics of the second SI reaction site and fruits with no seeds are found in this orchid group, further surprising us. What’s more, there seems to be more SI species with self-pollenated tube growth stopping at the base of column (Table 1), compared with *Dendrobium* species, which might suggest branch specificity. Therefore, a wider survey of the growth of pollen tube needs to be conducted, and the detailed changes in the shape of the pollen tube may also be examined, which may help answer questions on the phylogeny of SI in these groups.

### 2.3. Subtribe Oncidiinae

*Oncidiinae* has 65 genera and more than 1600 species [38,39]. Approximately 69.4% of *Oncidiinae* species are self-incompatible, 22.2% are self-compatible, and 8.3% have both self-incompatible and self-compatible populations [20]. The identification of SI in *Oncidiinae* is determined by fruit set and seed production after self-pollination [40]. In *Oncidiinae*, the growth of pollen tubes after self-pollination has only been reported in *Notylia nemorosa* (Table 1). The pollen tubes either did not germinate or stopped growing at the stigmatic surface, and the flowers withered approximately four days after pollination [31].

The high SI rate in this subtribe requires pollen tube observation experiments in more species, suggesting that different SI phenotypes exist in this group and that there might be another branch-specific trait.

### 2.4. Subtribe Aeridinae

The SI species reported in this subtribe is *Phalaenopsis pulcherrima.* Zhang’s research [41] on the breeding system of *P. pulcherrima* has shown that there is no significant difference in fruit set between self-pollination and cross-pollination, but the number of seeds with embryos in self-pollinated plants is significantly lower compared with cross-pollinated plants. From one to four days, the extent of pollen germination is lower and the length of the pollen tube is shorter after self-pollination. On the fifth day, there are fewer pollen tubes entering the ovule after self-pollination compared with cross-pollination [41]. These findings indicate that the SI of *P. pulcherrima* is late-acting SI before zygote formation. In this case, pollen can germinate, and pollen tubes can grow and enter ovules; however, the large number of self-pollinated fertilized eggs cannot develop into seeds [1,42].

Based on the above listed results, the SI phenotypes in orchids species contain each developmental stage from the beginning of hydration to the formation of the zygote after self-pollination, which is not reported in other angiosperm families. However, there are still a lot of pollination experiments to be done. For orchids groups with high SI rate, pollen tube observation experiments should be carried out in more species so that more SI phenotypes may be found, further verifying whether branch specificity exists among orchids groups. For species whose pollen tube growth stops at the stigma, reciprocal cross tests need to be performed in order to distinguish between GSI and SSI. In addition, in the past, many studies have evaluated the self-incompatibility of orchids based on fruit setting rate rather than pollen tube growth, and there has been no systematic mating system study on all orchids, so the proportion of self-incompatibility in orchids has been underestimated. A wider survey of mating systems based on pollen tube growth is needed, which will help to answer questions on the phylogeny of SI among orchids groups.

## 3. Physiology of SI in *Orchidaceae*

The pollen of the *Orchidaceae* contains large amounts of auxins [43]. Application of auxin to the stigma can result in post-pollination phenomena [44]. The use of auxin to treat self-incompatible orchids species can trigger flower abscission, but this can lead to parthenocarpy in self-compatible or partly self-compatible plants [18,45,46]. The detection results of the pollen substances of *Phalaenopsis* after pollination suggest that auxin, as the primary pollen signal [47], is transferred to the style and gradually infiltrates various organs, inducing ethylene production [48] and eventually leading to apoptosis in the perianth. The concentrations of auxin and ethylene were monitored during the development of pollen tube after self- and cross-pollination in *D.chrysanthum,* respectively [28]. The concentration of auxin was significantly higher within three days after self-pollination compared with cross-pollination. The concentration of ethylene (ACC) increased significantly at three days after self-pollination and decreased significantly at three days after cross-pollination. SI might fine-tune the auxin concentration, which promotes the production of ethylene [28], suggesting that auxin and ethylene might be involved in SI response.

However, further experiments are needed. Investigations into external hormone and hormone inhibitor treatment would be helpful to explore the effect of hormones on pollen tube development with self-pollination. Whether auxin and ethylene are involved in the SI response in other SI phenotypic species also needs to be verified. What is more, whether other hormones and ions, such as calcium involved in the SI response in *Papaveraceae* [3], are also involved in the orchids’ SI response, needs further verification.

## 4. Molecular Mechanisms of SI in *Orchidaceae*

Studies show that there are diverse SI phenotypes in orchids, even within the same genus, suggesting the high diversity and complex nature of the molecular mechanisms of SI that may exist in orchids. Since *S*-RNase-based GSI systems is observed in a wide range of flowering species [3], including Rosaceae, Solanaceae, and Plantaginaceae, and their SI phenotypes are similar to those of some orchids species, it is speculated that a similar molecular mechanism exists among them. Recently, based on orchids genome and transcriptome data, molecular identification of the *S*-RNase homolog in orchids was carried out, and revealed that no homologous gene was found in orchids [28,49]. The results suggest that SI is not mediated by *S*-RNase in orchids species, indicating new and undiscovered male and female determinants involving in orchids SI response. Furthermore, the WGCNA analysis based on transcriptome and hormone (auxin and ethylene) concentration data in *D. chrysanthum* revealed that there are some genes whose expression patterns are closely related to SI responses, such as genes encoding E3 ubiquitin-protein ligase, RING finger and CHY zinc finger domain-containing protein, NAC domain-containing protein, key enzymes in auxin and ethylene biosynthesis pathways, Ca^2+^-transporting ATPase, and the rapid alkalinization factors (RALF) [28]. Meanwhile, genes with pollen- and style-specific expression associated with SI were also screened out [28]. Then, based on functional annotation and interaction relationships between pollen- and style-specific expression genes, several SI closely related genes were considered to be candidate genes, including genes encoding ethylene biosynthesis enzyme, *RALF* genes and *CrRLK1L* genes [28], which is beneficial for the male and female determinants identification (Figure 1). So far, we are still a long way from understanding the molecular mechanisms of SI in orchids. However, it is feasible to identify what these genes do in orchids.

## 5. Challenges and Perspectives

Several morphological, physiological, and molecular features of SI in orchids have been studied. There are several pollen tube growth phenotypes in orchids, including the failure of pollinia to germinate, the cessation of pollen tube growth in the style, and the cessation of pollen tube growth in the ovary. Even within the same genus, such as in *Dendrobium*, there is more than one SI phenotypes, which is rarely reported in other angiosperm families in which SI molecular mechanisms are known. Furthermore, as one of the most diverse groups of angiosperms, SI is most common in the subfamily *Epidendroideae* of *Orchidaceae*, including *Dendrobium*, *Oncidiinae*, and *Pleurothaldinae*, which are groups containing large numbers of species, suggesting SI might be one of the main factors for promoting orchid species diversity. Physiological studies have suggested that auxin-induced ethylene production leads to premature flower abscission, which might be involved in the SI response. The molecular mechanisms of SI in orchids are complex and diverse and might differ from molecular mechanisms known in other angiosperm families. All these research levels are shown in Figure 1. The remarkable features prove that orchids are ideal materials for studying the molecular mechanisms and evolution of SI.

The study of SI in *Orchidaceae* is in an incipient stage. Several types of studies are needed to reveal the molecular mechanisms and the evolutionary process of SI in *Orchidaceae*. First, artificial pollination studies need to be conducted to facilitate the identification of phenotypes and haplotypes of SI in more orchids, especially by observing self- and cross-pollen tube germination and growth. Such work would enhance our understanding of the SI phenotype of orchids, and aid research examining the molecular mechanisms of SI. Second, SI-related loci and SI candidate genes need to be identified through genomic data analyses, such as genome and transcriptome sequencing, coupled with functional verification experiments; such work would not only enhance our understanding of the complex molecular mechanisms of SI in orchids but also aid hybrid breeding through the identification of advantagous haplotypes with high quality, especially for orchids with high market value, such as *Dendrobium* and *Phalaenopsis*. Third, transformation of corn poppy SI genes into *Arabidopsis thaliana* [50,51] has been shown to confer SI, and modification of SI genes in potato can make self-incompatible plants become self-compatible plants [52] suggesting that self-incompatible or self-compatible plants could be obtained through transformation or modification of the SI genes of specific orchids. With these techniques, new germplasm could be created by constructing artificial populations, such as F2, BC1, and RILs, which could aid the breeding of new varieties by selecting desired individuals.

In addition, the pollination strategy of *Orchidaceae* plants is widely known, and SI is common in large groups of orchids, such as, *Dendrobium*, and *Pleurothallidinae*. Therefore, the relationships among SI type, species number, flower morphology, and pollination strategy in the *Orchidaceae* phylogeny require clarification, as all of these characteristics contribute to the formation and maintenance of orchid species diversity. Most wild orchids are listed as endangered species, and all wild orchid species are included in the CITES (the Convention on International Trade in Endangered Species of Wild Fauna and Flora) appendix, which accounts for more than 90% of the plants protected by the Convention [53]. Studies on the relationship between SI and the formation and maintenance of population diversity will also provide new insights into the conservation biology of orchids.

## Figures and Tables

**Table 1:** Characteristics of the pollen tubes in orchids SI species.

Species	Site Where Pollen Tubes Stop Growing	Time When Pollen Tubes Stop Growing	Reference(s)
*D. longicornu* in Shenzhen	Stigma (absence or low pollen germination)		[28]
*D. cucullatum*			[30]
*Notylia nemorosa*			[31]
*Anathallis rubens* ^1^			[19,32]
*A. sclerophylla*			[19,32]
*A. heterophylla*			[19,32]
*Masdevallia infracta* ^2^			[19]
*Octomeria crassifolia* ^3^			[19]
*Octomeria grandiflora*			[19]
*Octomeria praestans*			[19]
*Stelis aff. Hypnicola* ^4^			[19]
*Stelis aff.peliochyla*			[19]
*Stelis* sp.			[19]
*Specklinia pristeoglossa* ^5^			[19]
*Specklinia* sp.			[19]
*D. densiflorum*	Beginning of the stylar channel (distal part of the column)	1 d	[28]
*D. thyrsiflorum*		1 d	[28]
*D. farmeri*		4 d	[18]
*D. moniliforme*		3 d	[28]
*D. catenatum*		2–3 d	[28]
*D. longicornu* in Yunnan		4 d	[28]
*D. chrysanthum*		3 d	[28]
*Acianthera saurocephala* ^6^		-	[19]
*D. unicum*	Stylar channel	4–5 d	[28]
*D. devonianum*		4–5 d	[28]
*D. denneanum*		4–5 d	[28]
*A. johannensis*	Stylar channel (base of the column)	7 d	[33]
*Acianthera adamantinensis*		-	[19]
*A. fabiobarrosii*		-	[19]
*Acianthera hamosa*		-	[19]
*Acianthera limae*		-	[19]
*Acianthera modestissima*		-	[19]
*Acianthera ochreata*		-	[19]
*Acianthera prolifera*		-	[19]
*Acianthera teres*		-	[19]
*Anathallis microphyta*		15 d	[32]
*Pleurothallis teres* ^4^		-	[34]
*D. lindleyi*	Before or after ovary entrance	2 d	[28]
*D. hancockii*		3–5 d	[28]
*D. jenkinsii*		3–5 d	[28]
*Restrepia brachypus* ^7^	Top third of ovary	21 d	[16]
*Pleurothallis johannensis*	In the ovary	-	[34]

The main clades of *Pleurothallidinae* according to Borba et al. (2011) [19]: ^1^ *Anathallis* belongs to clade D; ^2^ *Masdevallia* belongs to clade H; ^3^ *Octomeria* belongs to clade A; ^4^ *Stelis* and *Pleurothallis* belong to clade F; ^5^ *Specklinia* belongs to clade E; ^6^ *Acianthera* belongs to clade C; ^7^ *Restrepia* belongs to clade B. -: no data.
